# Cytochrome P450 F3 promotes colorectal cancer via inhibiting NRF2-mediated ferroptosis

**DOI:** 10.1016/j.tranon.2024.102077

**Published:** 2024-08-05

**Authors:** Ziyang Xu, Cheng Xu, Jie Lu, Chenfeng He, Xinyue Wang, Dongfei Zhu, Aizhong Wang, Zhengyun Zhang, Can Jiang

**Affiliations:** a The Department of Surgery, Shanghai Sixth People's Hospital Affiliated to Shanghai Jiao Tong University School of Medicine, Yishan Road 600, Shanghai 200233, China; b The Department of Anesthesiology, Shanghai Sixth People's Hospital Affiliated to Shanghai Jiao Tong University School of Medicine, Yishan Road 600, Shanghai 200233, China; c The Department of Integrative Bioanalytics, Aging and Cancer (IDAC), Institute of Development, Tohoku University, Sendai, Japan; d The Department of Investigative Pathology, Tohoku University Graduate School of Medicine, Sendai, Japan; e The Department of Cardiology, Shanghai Sixth People's Hospital Affiliated to Shanghai Jiao Tong University School of Medicine, Shanghai, China

**Keywords:** Colorectal cancer, Ferroptosis, CYP4F3, NRF2, Cytochrome P450

## Abstract

Cytochrome P450 F3 (CYP4F3) is recognized as a disease-associated immune response initiator that is involved in the synthesis of cholesterol, steroids, and lipids. This study identified the upregulation of *CYP4F3* expression in colorectal cancer (CRC) and its association with poor patient prognosis through a comparative analysis between CRC tumor tissues with normal tissues from public databases. The overexpression of *CYP4F3* in CT26.wt and SW620, promoted cell proliferation and migration, a reduction of cellular oxidative stress, an up-regulation of the oxidative stress-related pathway NRF2, and an inhibition of cellular ferroptosis. Additionally, inhibition of NRF2 activity stimulated cellular ferroptosis when *CYP4F3* was overexpressed. Ferroptosis, characterized by iron-dependent lipid peroxidation, is a non-apoptotic way of cell death with a critical role in cancer development. When given a ferroptosis agonist to CYP4F3-overexpression CRC cells, NRF2 was activated, and cell proliferation and migration were reduced. Furthermore, the mice subcutaneously injected with CYP4F3-overexpression CT26.wt cells formed significantly larger tumors compared to the CYP4F3-vector CT26.wt cell group. This study systematically identified an important role of *CYP4F3* in CRC development as a regulator of CRC cells to escape ferroptosis via NRF2, highlighting the significance of *CYP4F3* as a potential therapeutic target for CRC.

## Highlights

• Identification of CYP4F3 as a potential biomarker for CRC prognosis based on its upregulation in tumor tissues and association with poor patient survival.
• Elucidation of the role of CYP4F3 in promoting CRC cell proliferation and migration, accompanied by a reduction in cellular oxidative stress.
• Demonstration of the inhibitory effect of CYP4F3 on NRF2-mediated ferroptosis, highlighting its significance in CRC development and progression.
• Validation of our *in vitro* findings through *in vivo* experiments using a murine CRC model, further confirming the oncogenic role of CYP4F3 in CRC progression.

## Introduction

Colorectal cancer (CRC) is a widespread malignant tumor of the digestive tract, including colon cancer (COAD) and rectal cancer (READ) [1]. According to Cancer Statistics 2020, CRC is the third most prevalent cancer worldwide and ranks second among all cancers in terms of annual mortality [2]. Surgical intervention along with neoadjuvant medicines is currently the mainstay of CRC treatment, but the efficacy is unsatisfactory due to high metastasis and recurrence rates [3].

The mechanisms underlying the development and progression of CRC are complex and multifactorial. Ferroptosis is an iron-dependent and lipid peroxidation-driven cell death cascade, occurring when there is an imbalance of redox homeostasis in the cell. Recently, it has emerged as a crucial regulator of cancer progression and development [[4], [5], [6]]. Ferroptosis is induced by three mechanisms: accumulation of lipid peroxides, suppression of glutathione peroxidase 4 (GPX4) activity, and depletion of intracellular glutathione (GSH) [5,7]. *NRF2* (nuclear factor erythroid 2-related factor 2, NFE2L2) is key to the cellular antioxidant response and highlighting its pivotal role in modulating sensitivity to ferroptosis [8]. Therefore, investigating the responsiveness of manipulating the NRF2 signaling pathway to regulate ferroptosis holds significant importance in identifying innovative therapeutic strategies for CRC.

Cytochrome P450 F3 (CYP4F3), involved in synthesizing cholesterol, steroids, and lipids, has been recognized as an immune response initiator in diseases [9]. It is a key molecule in inactivating and degrading leukotriene B4 and regulating arachidonic acid metabolism [10]. Moreover, Gene Ontology annotations related to it include iron ion binding and oxidoreductase activity, acting on paired donors, with incorporation or reduction of molecular oxygen [11]. Recent studies have shown the involvement of *CYP4F3* in tumor development. For instance, Wang et al. identified *CYP4F3* as a prognostic marker for endometrial cancer [12], while Jia et al. identified it as a key gene in the TF gene network of osteosarcoma [13]. Zhao et al. also found its influence on the tumor microenvironment in lung cancer [14]. Furthermore, *CYP4F3* was identified as one of the 117 common genes up-regulated in cold tumors across smoking-related cancers, exerting dominance in immunoregulation and associated with NRF2 pathway activation and the tumor immune microenvironment [15]. However, the role of *CYP4F3* in regulating CRC development remains elusive.

In this study, *CYP4F3* was found to be highly expressed in CRC. The overexpression of *CYP4F3* enhanced the resistance of CRC cells to ferroptosis by upregulating NRF2 and promoted tumor proliferation and migration. Thus, *CYP4F3* may play a crucial role in regulating the sensitivity of CRC cells to ferroptosis, which indicates its potential as a therapeutic target for CRC.

## Materials and methods

### Bioinformatics analysis

GEPIA2 (http://gepia2.cancer-pku.cn/, accessed on December 4, 2023), UALCAN database (https://ualcan.path.uab.edu/, accessed on December 4, 2023) and TCGA database (https://portal.gdc.cancer.gov/, accessed on December 4, 2023) were utilized to conduct a comparative analysis of *CYP4F3* expression between CRC and normal tissues [16,17]. Concurrently, the impact of *CYP4F3* on both OS and DFS among patients were accessed by the Kaplan-Meier Plotter (https://kmplot.com/analysis/, accessed on December 4, 2023). Furthermore, UALCAN was used to validate *CYP4F3* expression in CRC and its correlation with *tp53* mutations and N-staging. R (version 4.3.1) and its packages were used within RStudio to process data from TCGA.

### Patient samples

Patient biopsy samples were collected at the Shanghai Sixth People's Hospital Affiliated to Shanghai Jiao Tong University School of Medicine between 2022.12 and 2023.12. Patients who underwent preoperative treatments, such as radiotherapy or chemotherapy were excluded from the study. The patients were classified into three groups: normal, intraepithelial neoplasia, and adenocarcinoma. These specimens were subjected to hematoxylin-eosin staining and immunostaining for Ki67 and CYP4F3. This study was approved by the Ethics Committee of Shanghai Sixth People's Hospital Affiliated to Shanghai Jiao Tong University School of Medicine.

### Cell line and *in vitro* transfection

CT26.wt, a murine colorectal carcinoma cell line, and SW620, a human colorectal carcinoma cell line, were purchased from the Cell Bank of Chinese Academy of Sciences (Shanghai, China). CT26.wt cells were cultured in RPMI 1640 medium (MA0315, Meilun) supplemented with 10 % fetal bovine serum (PWL001, Meilun) and 1 % penicillin/streptomycin (MA0347, Meilun). SW620 cells were cultured in Leibovitz's l-15 medium (11,415,064, Gibco) supplemented with 10 % fetal bovine serum (PWL001, Meilun) and 1 % penicillin-streptomycin (MA0347, Meilun). Both cell lines were incubated at 37 °C in a 5 % carbon dioxide/water-saturated incubator.

The transfection experiments utilized the pLV3-CMV-CYP4F3-CopGFP-Puro plasmid obtained from Miaoling Biology (Wuhan, China), and the *CYP4F3* gene ID is NM_000896.3. Plasmid extraction kit from Tiangen Biotech (DP104, Beijing, China). The construction of stable cell lines followed the protocol described by Tandon et al. [18]. Prior to transfection, the target plasmid was mixed with polyethyleneimine (PEI) at a mass ratio of 1:3 to form DNA-PEI complexes in DMEM medium. After 48 h of infection, the supernatant was collected and added to CT26.wt and SW620 cells for infection, named CT26.wt-OE (CYP4F3 overexpression) and SW620-OE (CYP4F3 overexpression). After 5 days of culture, 2 μg/ml of puromycin was added for selection. The control group was transfected with an empty vector for construction, named CT26.wt-NC (negative control) and SW620-NC (negative control). Transfection efficiency was assessed by capturing images with a fluorescence microscope. Cell migration was assessed by the wound healing experiment, followed by photographic documentation. The cells were collected and evaluated for cell proliferation using the CCK-8 kit (MA0218, Meram). Intracellular MDA and GSH/GSSG ratio levels were quantified using a Lipid Peroxidation MDA Assay Kit (S0131, Beyotime Biotechnology, Beijing, China) and GSH/GSSG Ratio Detection Assay Kit II (ab205811, Abcam). Detection of Fe^2+^ content using Cell Ferrous Iron (Fe^2+^) Fluorometric Assay Kit (MA0647, Meilun).

### Erastin treatment and inhibitor treatment

CT26.wt-NC/OE and SW620-NC/OE cells were treated with 20 μM Erastin (HY-15,763, MedChemExpress), a ferroptosis agonist. After treatment for 24 h, cell proliferation and migration were measured.

To inhibit NRF2 activity, CT26.wt-NC/OE and SW620-NC/OE cells were treated with 3 μM ML385 (HY-100,523, MedChemExpress) for 2 h. Intracellular MDA and GSH/GSSG ratio levels were quantified.

### RT-qPCR analysis

Total RNA was isolated from cells using the EZ-press RNA Purification Kit (B0004D, EZBioscience, USA). The extracted RNA was reverse transcribed into cDNA using the 4 × EZscript Reverse Transcription Mix II (with gDNA Remover) (EZB-RT2GQ, EZBioscience, USA). RT-qPCR analysis was performed using the 2 × SYBR Green qPCR Master Mix (A0001, EZBioscience, USA). The RNA concentration was determined using the Nanodrop2000 spectrophotometer (Thermo Fisher Scientific). GAPDH was utilized as an internal reference gene for normalization and calculation of fold changes in mRNA expression levels relative to control samples. Primer sequences are provided in the following Supplemental Table 1.

### Animal and tumor induction

Experimental animals used in this study were 6-week-old Balb/c mice purchased from spfbiotech (Beijing, China). Mice were housed at 23 °C ± 2 °C in a 12 h light/dark cycle and supplied with food and water with free access at the animal facilities of Shanghai Sixth People's Hospital affiliated with Shanghai Jiao Tong University School of Medicine (Shanghai, China). All animal experiments were approved by the Ethics Committee of Shanghai Sixth People's Hospital affiliated with Shanghai Jiao Tong University School of Medicine.

CT26.wt-NC and CT26.wt-OE were collected and counted by a cell counting chamber. Each mouse received a subcutaneous injection of 200 million cells to induce tumor formation. After 21 days of feeding, mice were euthanized, and tumors were collected. During the experiment, regular examinations of tumor growth in the animals were conducted, and tumor diameters were recorded. Once the tumor diameter exceeded 15 mm, euthanasia was performed.

### Pathological analysis, immunohistochemistry, and immunofluorescence staining

Tumors collected from mice were rapidly fixed in 4 % paraformaldehyde and embedded in paraffin. A series of sections (4 mm thick) of the formalin-fixed, paraffin-embedded tissues were stained with hematoxylin-eosin.

Immunohistochemical staining of formalin-fixed, paraffin-embedded samples was conducted using the following primary antibodies; rabbit anti-CYP4F3 (1:100, bs-14160R, Bioss, Beijing, China.), rabbit anti-NRF2 (1:300, 16,396–1-AP, Proteintech, Wuhan, China), rabbit anti-Ki67 (1:200, ab16667, Abcam, Cambridge, England), and mouse anti-GPX4 (1:1000, 67,763–1-Ig, Proteintech, Wuhan, China) with Histofine Simple Stain MAX-PO (Nichirei Biosciences, Tokyo, Japan), following the manufacturer's protocol.

Immunofluorescence staining of paraffin-embedded samples was performed using anti-CYP4F3 (1:100), anti-NRF2 (1:300), anti-Ki67 (1:200), anti-GPX4 (1:1000) and HRP secondary antibody (1:2000). All stained sections were scanned using Pannoramic MIDI (3DHISTECH Ltd). The pathological support was provided by Ningbo Yangming Medical Inspection Laboratory Co., Ltd.

### Immunoblotting analysis

Total proteins were exacted from cells by RIPA lysis buffer (G2002–100 ML, Servicebio, Wuhan, China) supplemented with 1 % PMSF (G2008–1 ML, Servicebio, Wuhan, China) and 1 % phosphatase inhibitor (G2007–1 ML, Servicebio, Wuhan, China). Protein concentration was used the BCA protein concentration assay kit (G2026–200T, Servicebio, Wuhan, China),

The extracted proteins underwent separation via 10 % SDS-PAGE (G2043–50T, Servicebio, Wuhan, China) and were subsequently transferred onto a PVDF membrane (0.45 μm, G6015–0.45, Servicebio, Wuhan, China). The PVDF membranes were subjected to overnight incubation at 4 °C with the corresponding primary antibodies to ensure robust binding. Incubation with secondary antibodies was carried out for 2 h at room temperature. Upon completion of the incubation period, the membrane was immersed in ECL luminescent solution (G2161–200ML, Servicebio, Wuhan, China) for chemiluminescence. Bound antibodies were visualized in the ImageQuant LAS 4000 (GE Healthcare, Chicago, IL) and quantified by ImageJ software version 1.53 (NIH, Bethesda, MD; https://imagej.nih.gov). The following antibodies were used: rabbit anti-CYP4F3 (1:1000, bs-14160R, Bioss, Beijing, China.), rabbit anti-NRF2 (1:2000, 16,396–1-AP, Proteintech, Wuhan, China), rabbit anti-Ki67 (1:1000, ab16667, Abcam, Cambridge, England), mouse anti-GPX4 (1:3000, 67,763–1-Ig, Proteintech, Wuhan, China), anti-mouse IgG (1:2000; NA931V; Cytiva, Tokyo, Japan), and anti-rabbit IgG (1:2000; number 7074S; Cell Signaling Technology Inc.).

### Statistical analysis

All data are expressed as means ±SD. The differences between two groups were analyzed using a two-tailed *t*-test, and differences between three or more groups were analyzed using a two-way ANOVA followed by a *Tukey post hoc test. p < 0.05* was considered statistically significant. All statistical analyses were performed using GraphPad Prism 8 for Windows (GraphPad Software, Boston, Massachusetts USA, www.graphpad.com).

## Results

### *CYP4F3* is upregulated in CRC tumor tissues

**Fig. 1:**
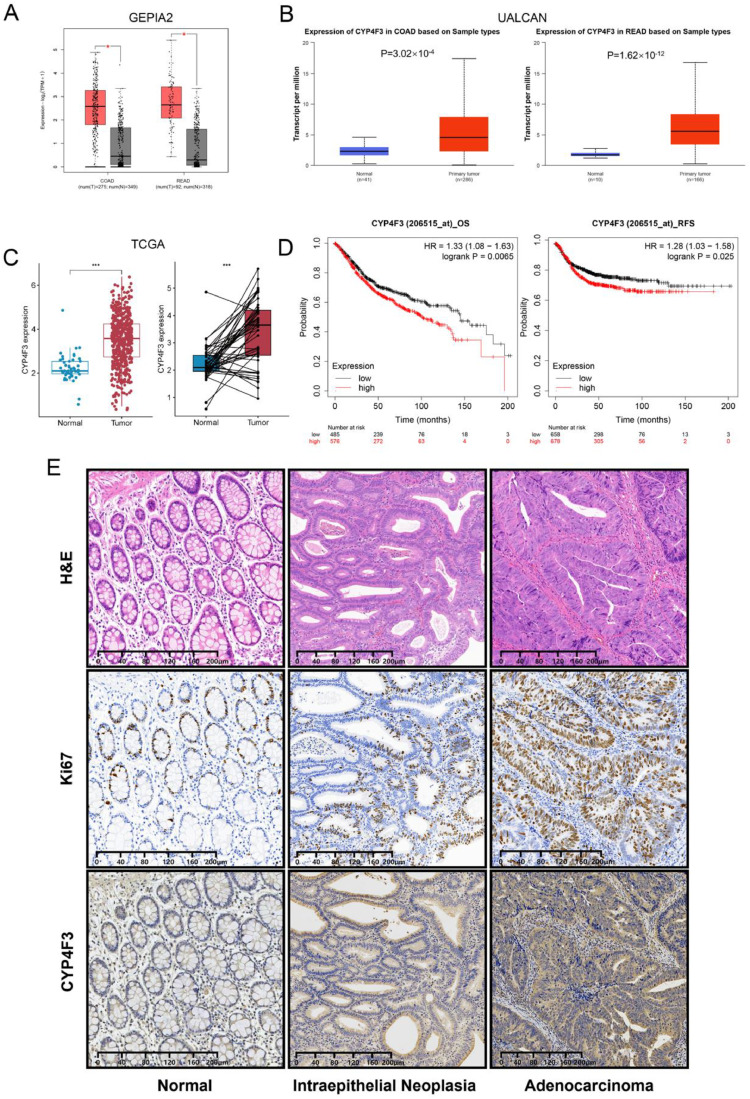
*CYP4F3* expression is up-regulated in CRC. A-C. High expression of CYP4F3 in COAD and READ tissues compared to normal intestinal tissues in GEPIA2, UALCAN, and TCGA databases; D. Patients with high CYP4F3 expression show poorer OS and RFS according to the KM plotter database; E. Representative hematoxylin-eosin (H&E) staining and immunohistochemistry (IHC) images of CYP4F3 and Ki67 in CRC patients’ samples. From left to right, the images show normal intestinal mucosa epithelium, intraepithelial neoplasia, and colorectal carcinoma tissues. The expression of CYP4F3 increases with the proliferation marker Ki67.The data are expressed as means ±SD. Original magnification, × 100 (E). (*p<0.05; ** p<0.01; *** p<0.001).

A comparative analysis of gene expression between COAD and READ tumor tissues and normal tissues was performed using GEIPA2 (http://gepia2.cancer-pku.cn/) [16], UALCAN (https://ualcan.path.uab.edu/) [17] and TCGA (https://portal.gdc.cancer.gov/). *CYP4F3* expression was found to be higher in CRC tissues compared to normal tissues (Fig. 1A–C), and there was a correlation between the expression level and the presence of *TP53* mutation (Supplemental Fig. S1). Following this, we found patients with high level of *CYP4F3* expression had considerably lower rates of overall survival (OS) and recurrence free survival (RFS) by using the Kaplan Meier plotter database (https://kmplot.com/analysis/) [19] (Fig. 1D). Similarly, immunostaining revealed that *CYP4F3* expression was higher in patients’ tumor tissues compared to intraepithelial neoplasia and normal tissues (Fig. 1E). Therefore, *CYP4F3* may enhance the CRC progression.

### *CYP4F3* promotes CRC cell proliferation and migration

**Fig. 2:**
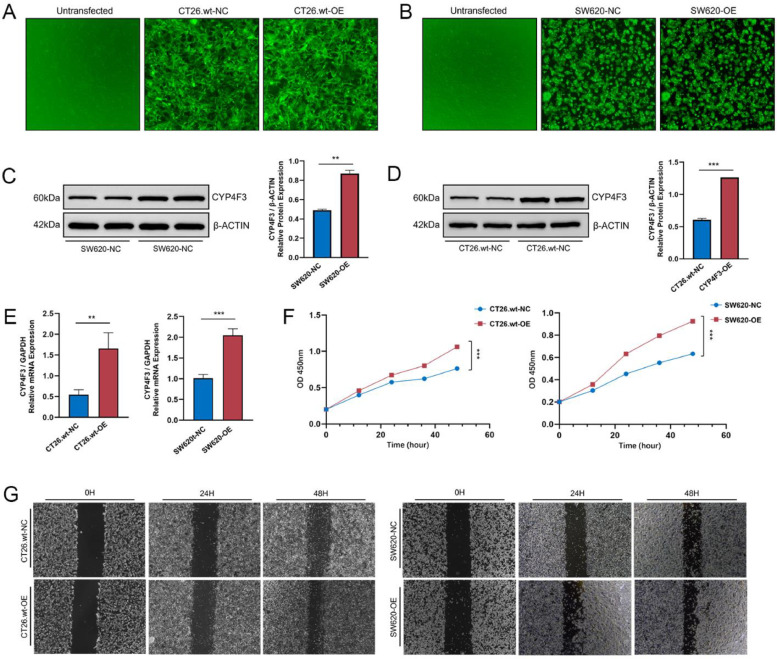
*CYP4F3* promotes CRC cells proliferation and migration. A-E. Validation of CYP4F3 expression levels by fluorescence microscope images (A, B), WB (C, D) and RT-qPCR (E); F-G. CCK-8 assay (F) and scratch wound-healing assay (G) of CT26.wt-OE/NC and SW620-OE/NC cells. The data are expressed as means ±SD. *n* = 2 in per groups (C-D); *n* = 6 in per group (E-F). Original magnification, × 400 (A, B, G) (** p<0.01; *** p<0.001).

To investigate the potential role of *CYP4F3* on CRC cell proliferation and migration, we established a stable overexpression of *CYP4F3* in the CT26.wt and SW620 cell line, denoted as CT26.wt-OE and SW620-OE, along with its corresponding control, CT26.wt-NC and SW620-NC, which was established using empty vector construct. Images captured by fluorescence microscope demonstrate excellent transfection efficiency (Fig. 2A, B). Additionally, the results of WB and RT-qPCR confirm the excellent transfection efficiency of the both cells (Fig. 2C–E). Subsequently, Cell Counting Kit-8 (CCK-8) assay was measured to evaluate cell proliferation and scratch wound-healing assay was used to assess cell migration in response to *CYP4F3* overexpression. As depicted in Fig. 2F, CT26.wt-OE and SW620-OE significantly augmented CRC cell proliferation. Moreover, the scratch wound-healing assay revealed that CT26.wt-OE and SW620-OE exhibited increased migratory ability compared to CT26.wt-NC and SW620-NC (Fig. 2G). These results suggest *CYP4F3* may enhance the CRC cell proliferation and migration.

### *CYP4F3* attenuates oxidative stress *in vitro*

**Fig. 3:**
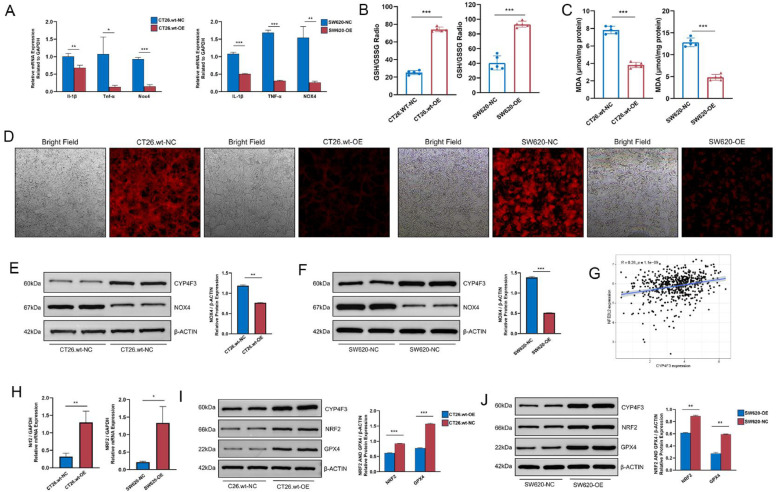
*CYP4F3* inhibits ferroptosis in CRC cells. A. RT-qPCR analysis of oxidative stress molecules (*IL-1β, TNF-α*, and *NOX4*) of CT26.wt-OE/NC and SW620-OE/NC cells; B-C. GSH/GSSG (B) and MDA levels (C) in CT26.wt-OE/NC and SW620-OE/NC cells; D. Fluorescence microscope images of cellular Fe^2+^; E-F. Western blot analysis of NOX4 of CT26.wt-OE/NC and SW620-OE/NC cells; G. Positive correlation between CYP4F3 expression and NRF2 in CRC transcriptome data from TCGA; H. RT-qPCR analysis of NRF2 expression of CT26.wt-OE/NC and SW620-OE/NC cells; I-J. Western blot analysis of CYP4F3, NRF2 and GPX4 in CT26.wt-OE/NC and SW620-OE/NC cells. The data are expressed as means ±SD. *n* = 2 in per groups (E, H, I, J); *n* = 6 in per group (A-C, H). Original magnification, × 1000 (D) (*p<0.05; ** p<0.01; *** p<0.001).

To clarify the potential role of *CYP4F3* in CRC, the expression levels of oxidative stress-related molecules, *IL-1β, TNF-α*, and *NOX4*, were examined in CT26.wt-OE/NC and SW620-OE/NC cells. CT26.wt-OE and SW620-OE cell showed marked lower expression levels of *IL-1β, TNF-α*, and *NOX4* compared to CT26.wt-NC and SW620-NC cell by RT-qPCR and analysis (Fig. 3A). The levels of malondialdehyde (MDA) and the ratio of GSH to oxidized glutathione (GSSG) in the two cell groups were evaluated, where MDA serves as a measure of lipid peroxidation and the GSH/GSSG ratio reflects the degree of oxidative damage within the cells. Remarkably, CT26.wt-OE and SW620-OE cell showed a decrease of MDA levels, accompanied by an increase of the GSH/GSSG ratio, indicating that the overexpression of *CYP4F3* may contribute to reducing intracellular oxidative stress, thereby decreasing the production of lipid peroxidation and increasing the availability of reduced glutathione, thus enhancing the antioxidant capacity of CRC cells (Fig. 3B, C). The results from the Cell Ferrous Iron (Fe^2+^) detection assay also indicated that CRC cells overexpressing CYP4F3 exhibited lower intracellular Fe^2+^ levels (Fig. 3D). Moreover, immunoblotting analysis of NOX4 showed similar results in both CT26.wt-OE/NC and SW620-OE/NC cells (Fig. 3E-F).

A close relationship between the expression of *CYP4F3* and *NRF2* was observed in CRC tumor tissues (Fig. 3G), whereas, in contrast, there is no significant association between them in normal tissues from public databases (Supplemental Fig. S1). Therefore, we evaluated the expression of *NRF2* between the two cell groups and simultaneously measured the expression levels of *GPX4*. The significantly increased expression levels of *NRF2* and *GPX4* were found in CT26.wt-OE and SW620-OE cell (Fig. 3H-J). These findings suggest that *CYP4F3* may enhance the resistance of CRC cells to oxidative stress and upregulate *NRF2* expression, which alleviates ferroptosis in CRC cells.

### *CYP4F3* attenuates cellular ferroptosis

**Fig. 4:**
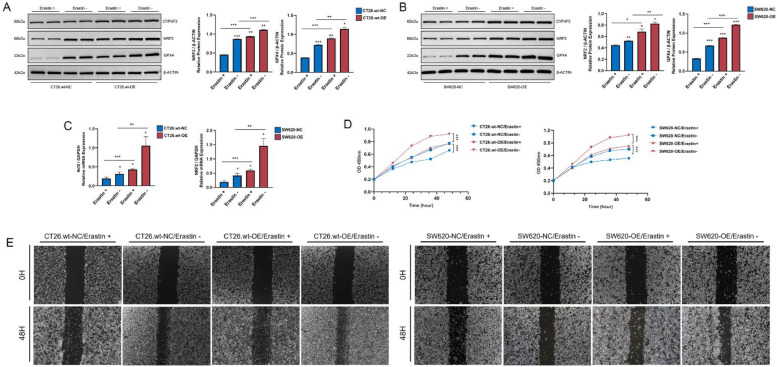
Erastin reduces ferroptosis resistance in CRC cells overexpressing *CYP4F3*. A-B. Western blot analysis of CYP4F3, NRF2 and GPX4 in CT26.wt-OE/NC and SW620-OE/NC cells with or without Erastin treatment; C. RT-qPCR analysis of NRF2 after Erastin intervention; d-E. Effects of Erastin intervention on the cell proliferation and migration assessed by CCK8 (D) and scratch wound-healing assay (E). The data are expressed as means ±SD. *n* = 2 in per groups (A-B); *n* = 6 in per group (C-D). Original magnification, × 400 (E). (*p<0.05; ** p<0.01; *** p<0.001).

To elucidate the regulatory role of *CYP4F3* on cellular ferroptosis, CT26.wt-OE/NC and SW620-OE/NC cells were treated with Erastin (MCE, HY-15,763, 20 μM), a ferroptosis agonist, to stimulate cellular ferroptosis. Erastin treatment inhibited the activation of NRF2 and decreased *GPX4* expression in CT26.wt-OE/NC and SW620-OE/NC cells. The expression levels of *NRF2* and *GPX4* in the Erastin-treated CT26.wt-OE and SW620-OE cells were comparable to those in the untreated CT26.wt-NC and SW620-NC cells (Fig. 4A-C). Furthermore, the results of the CCK-8 assay and scratch wound-healing assay demonstrated that Erastin treatment suppressed the cell proliferation and migration. The ranking of cell proliferation and migration rates from weakest to strongest is as follows: CT26.wt-NC/Erastin+ and SW620-NC/Erastin+, CT26.wt-NC/Erastin- and SW620-NC/Erastin-, CT26.wt-OE/Erastin+ and SW620-OE/Erastin+, and CT26.wt-OE/Erastin- and SW620-OE/Erastin- (Fig. 4D, E). These results indicate that the inhibition ferroptosis by *CYP4F3* is nearly reversed by Erastin treatment, and this process may be mediated by NRF2.

### Inhibition of NRF2 promotes cellular ferroptosis

**Fig. 5:**
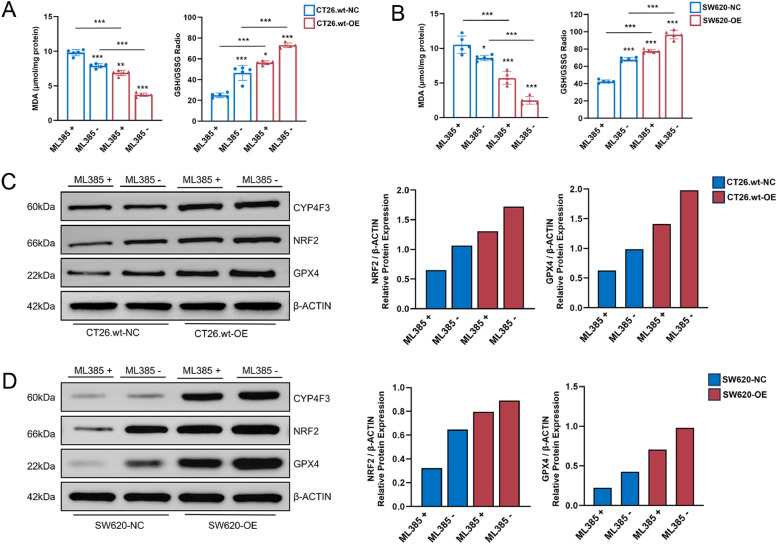
Inhibition of NRF2 reduces ferroptosis. A-B. Levels of MDA and GSH/GSSG ratio in cells treated with or without ML385; C-D Western blot analysis of CYP4F3, NRF2 and GPX4 in CYP4F3-OE/NC and SW620-OE/NC cells with or without ML385 treatment. The data are expressed as means ±SD. *n* = 2 in per groups (C-D); *n* = 6 in per group (A-B). (*p<0.05; ** p<0.01; *** p<0.001).

To investigate the role of NRF2 in regulating ferroptosis, CT26.wt-OE/NC and SW620-OE/NC cells were treated with ML385 (MCE, HY-100,523, 3 μM), an NRF2 inhibitor known to suppress NRF2 transcriptional activity by impacting the DNA binding activity of the NRF2-MAFG protein complex. The addition of ML385 led to an elevation of intracellular MDA levels and a reduction of the GSH/GSSG level in both CT26.wt-OE/NC and SW620-OE/NC cells. Moreover, these changes were more pronounced in CT26.wt-OE and SW620-OE cells (Fig. 5A, B). An immunoblotting analysis was conducted to assess alterations in *GPX4* expression (Fig. 5C, D), revealing a reduced level of *GPX4* expression in cells treated with ML385. These findings collectively suggest that CYP4F3-induced ferroptosis may be mediated through upregulation of NRF2.

### *CYP4F3* promotes CRC cell proliferation *in vivo*

**Fig. 6:**
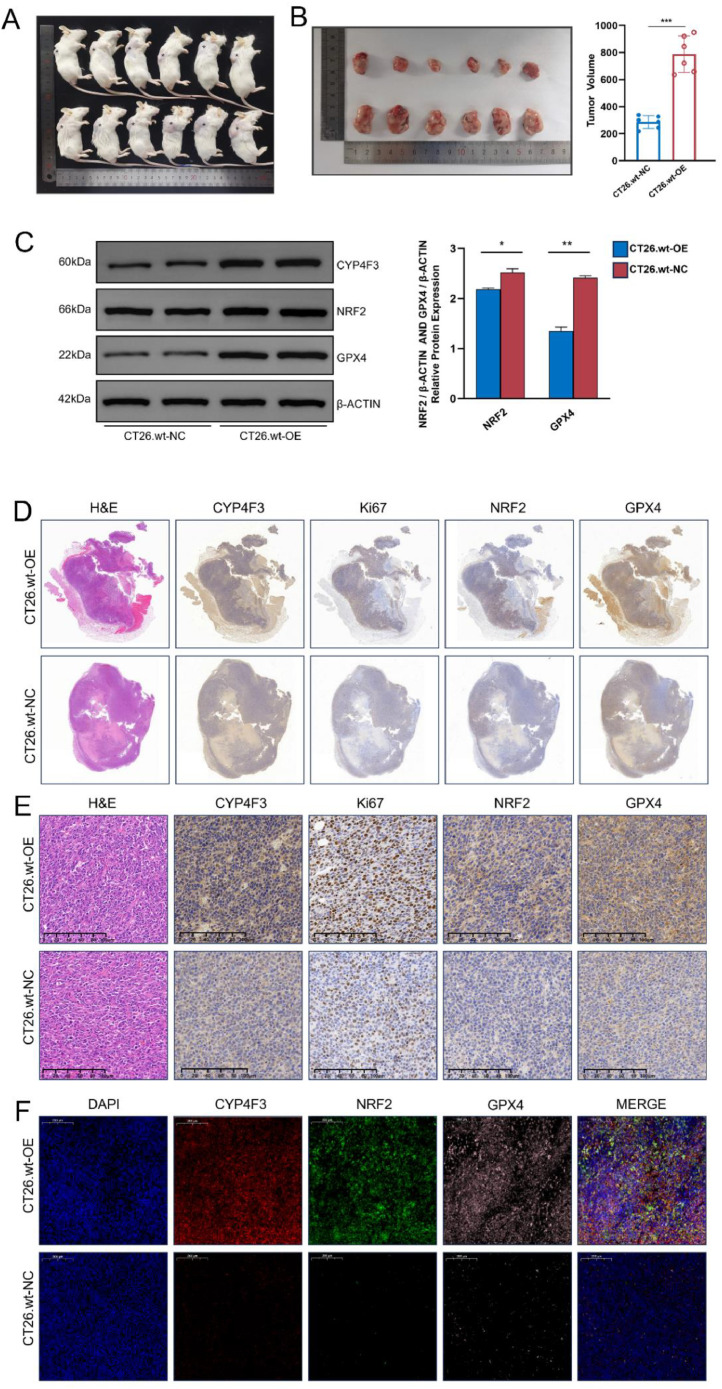
*CYP4F3* promotes cell proliferation in CRC through NRF2-GPX4 axis. A-B. Subcutaneous tumor formation in Balb/c mice after 21 days of normal feeding (* indicates tumor location). Macroscopic image of mice (A) and comparison of formed tumors and tumor volumes (B).; C. Western blot analysis of CYP4F3, NRF2 and GPX4 in CT26.wt-OE and CT26.wt-NC groups; d-E. Representative hematoxylin-eosin (H&E) staining and immunohistochemistry (IHC) images of CYP4F3, Ki67, NRF2, and GPX4 in Balb/c mouse tumors; F. Immunofluorescence staining images of CYP4F3, NRF2, and GPX4 in Balb/c mouse tumors. The data are expressed as means ±SD. *n* = 6 in per group (A, B); *n* = 2 in per groups (C). Original magnification, × 100 (D). Original magnification, × 400 (E, F). (*** p<0.001).

To validate the exact role of *CYP4F3 in vivo*, subcutaneous tumor formation models were established in BALB/c mice by injecting CT26.wt-OE and CT26.wt-NC cells. After 21 days, the mice were euthanized, and the subcutaneous tumors were harvested. The tumor size was significantly larger in the CT26.wt-OE group compared to the CT26.wt-NC group (Fig. 6A, B). The expression levels of *NRF2* and *GPX4* in the CT26.wt-OE group were significantly elevated (Fig. 6C). Furthermore, immunostaining of *CYP4F3, Ki67, NRF2*, and *GPX4* were performed on the tumor tissues. The panoramic view and local details are shown in Fig. 6D, E. In the CT26.wt-OE group, up-regulation of *Ki67* expression was predominantly found in the nuclear, and *NRF2* and *GPX4* expression were up-regulated in the cytoplasm compared to the CT26.wt-NC group. In addition, the results of the immunofluorescence staining also corroborated these findings (Fig. 6F). These findings reconfirm that *CYP4F3* promotes CRC progression, which is achieved by upregulating NRF2 to inhibit the cellular ferroptosis.

## Discussion

**Fig. 7:**
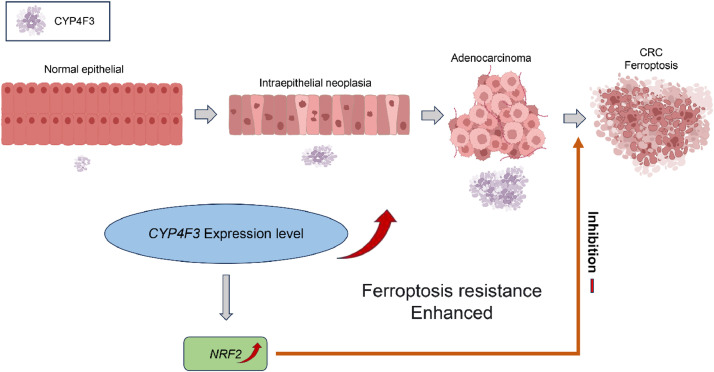
Schematic representation of the mechanism by which *CYP4F3* inhibits NRF2-mediated ferroptosis to promote CRC progression.

This study demonstrates that *CYP4F3* efficiently promotes CRC through the upregulation of *NRF2* and reduction of cell susceptibility to ferroptosis. The inhibition of NRF2 was effective in enhancing cellular ferroptosis. Therefore, *CYP4F3* plays a pivotal role in tumor development and progression through obstructing NRF2-mediated ferroptosis (Fig. 7).

NRF2 is a bZIP redox-sensitive transcription factor that activates the expression of target genes in response to cellular stresses by binding to antioxidant response elements (AREs) in their promoters [20]. NRF2 has multiple effects on cancer, promoting tumor proliferation, counteracting oxidative stress, and regulating drug metabolism [[21], [22], [23]]. When tumor cells proliferate rapidly, insufficient angiogenesis leads to intratumoral hypoxia, exhibiting a high metabolic rate and elevated ROS level, which in turn promotes cell death [24]. In this circumstance, NRF2 is aberrantly activated to maintain intracellular redox homeostasis, resist oxidative stress injury, and promote cell proliferation to counteract cell death [25,26]. In this study, the overexpression of *CYP4F3* activated NRF2 and the inhibition of NRF2 increased MDA levels and reduced the GSH/GSSG level in CRC cells. Oxidative stress plays an important role in ferroptosis [5]. Ferroptosis is an excessive lipid peroxidation-induced cell death that can suppress tumor invasion [27]. NRF2 is a key regulator in ferroptosis owing to its regulatory role in oxidative stress [20]. We found that Erastin treatment suppressed cell proliferation and migration. *CYP4F3* overexpression upregulated *GPX4* expression and inhibition of NRF2 downregulated *GPX4* expression in CRC cells. *GPX4* is a major ferroptosis regulator that uses glutathione to protect cells from ferroptosis by eliminating phospholipid peroxides [28]. We also observed that NRF2 activity was inhibited after ferroptosis agonist treatment. Thus, the results suggest that *CYP4F3* promotes cell proliferation and migration in CRC by inhibiting NRF2-mediated ferroptosis.

*CYP4F3* oxidizes arachidonic acid, producing 20-HETE, and ω-hydroxylates very long-chain fatty acids, thereby playing a role in the regulation of oxidative stress [[29], [30], [31]]. Zhang et al. found that overexpression of CYP450 induces ubiquitination, which promotes NRF2 translocation to the nucleus to exert antioxidant effects [32]. In our study, *CYP4F3* overexpression reduced intracellular oxidative stress, resulted in larger subcutaneous tumors and upregulated *GPX4* and NRF2 expressions in mice. *Ki67* intense immunostaining in *CYP4F3* overexpression tumors suggests that *CYP4F3* promotes tumor progression. Wen et al. showed that *CYP4F3* increased arachidonic acid and ROS accumulation, leading to ferroptosis, which promoted hepatocellular carcinoma progression [33]. This contradicts our findings, and the reasons for this discrepancy are currently unknown. Although it could be attributed to the organ-specificity of the regulatory effects of *CYP4F3* on tumors, this discrepancy deserves further research. J Costa et al. reported that the *CYP4F3*/20-HETE pathway adversely affected the cardiovascular system in ovariectomized hypertensive women [34]. Alexanian et al. also noted that CYP4F produced large amounts of 20-HETE, which caused the proliferation of renal carcinoma cells [35]. Furthermore, it has been shown that 20-HETE activates NADPH oxidase and produces NADPH oxidase-derived superoxide, which provides cells with a survival advantage and protects them from death [36,37]. However, whether *CYP4F3* regulates NRF2 expression through 20-HETE is currently unknown and should be further investigated. Nevertheless, this study indicates the crucial role of the CYP4F3-NRF2 axis in CRC.

This study has several limitations. First, the clinical samples collected were insufficient to improve the credibility of this study. In addition, studying the therapeutic potential of a pharmacologic inhibitor of *CYP4F3* in attenuating CRC progression is essential.

In conclusion, this study employed both human and murine cell lines for *in vitro* and *in vivo* experiments, elucidating the role of *CYP4F3* in regulating CRC progression, predominantly through regulating NRF2-mediated ferroptosis. Currently, targeting NRF2 and promoting ferroptosis for cancer treatment is a highly scrutinized direction [20,38]. However, directly inhibiting NRF2 is challenging while targeting NRF2 upstream factors may hold more promise [39]. This study highlights the potential of *CYP4F3* to be a specific target for the prevention and treatment of CRC.

Supplemental Fig. S1. Relationship of CYP4F3 with TP53 Mutation and NRF2 Expression in CRC. A-B. Association between CYP4F3 expression and TP53 mutation status in CRC; C. Analysis using GEPIA2 reveals a positive correlation between CYP4F3 expression and NRF2 levels in CRC tumor tissues from TCGA dataset; D. Analysis using GEPIA2 demonstrates a significant negative correlation between CYP4F3 expression in normal intestinal tissues from TCGA and GTEx datasets and NRF2 expression.

## Ethics approval and consent to participate

The study was approved by the Ethics Committee of Shanghai Sixth People's Hospital Affiliated to Shanghai Jiao Tong University School of Medicine. Informed consent was obtained from all subjects involved in the study. The animal study protocol was approved by the Ethics Committee of Shanghai Sixth People's Hospital affiliated with Shanghai Jiao Tong University School of Medicine. All procedures adhered to the ethical principles of medical research involving human subjects as outlined in the 1964 Helsinki Declaration and its subsequent amendments for ethical research involving human subjects.

## Consent for publication

The article is original, has not already been published in a journal, and is not currently under consideration by another journal. All authors of the manuscript have read and agreed to its content for publication.

## Funding

This research was funded by Shanghai Sixth People's Hospital affiliated to 10.13039/501100004921Shanghai Jiao Tong University School of Medicine (grant number ynts202209) and the Postdoctoral Fellowship Program of 10.13039/501100002858China Postdoctoral Science Foundation (grant number 2024M752026).

## Ethical Statement

The animal and human study protocols were approved by the Ethics Committee of Shanghai Sixth People's Hospital Affiliated to Shanghai Jiao Tong University School of Medicine. All procedures adhered to the ethical principles of medical research involving human subjects as outlined in the 1964 Helsinki Declaration and its subsequent amendments for ethical research involving human subjects.

## Informed Consent Statement

Informed consent was obtained from all subjects involved in the study.

## CRediT authorship contribution statement

**Ziyang Xu:** Conceptualization, Investigation, Methodology, Resources, Data curation, Writing – original draft. **Cheng Xu:** Conceptualization, Investigation, Methodology, Resources, Data curation, Funding acquisition. **Jie Lu:** Conceptualization, Investigation, Methodology, Resources, Data curation. **Chenfeng He:** Software, Visualization, Formal analysis, Resources, Data curation. **Xinyue Wang:** Software, Visualization, Formal analysis, Resources, Data curation. **Dongfei Zhu:** Software, Visualization, Formal analysis, Resources, Data curation. **Aizhong Wang:** Supervision, Project administration, Validation, Writing – review & editing. **Zhengyun Zhang:** Supervision, Project administration, Validation, Writing – review & editing. **Can Jiang:** Conceptualization, Investigation, Methodology, Supervision, Project administration, Validation, Writing – review & editing.

## Declaration of competing interest

The authors declare no competing interest.

## Appendix Supplementary materials

Image, application 1

Image, application 2

## References

[1] Araghi M., Arnold M., Rutherford M.J. (2021). Colon and rectal cancer survival in seven high-income countries 2010-2014: variation by age and stage at diagnosis (the ICBP SURVMARK-2 project). Gut.

[2] Sung H., Ferlay J., Siegel R.L. (2021). Global cancer statistics 2020: GLOBOCAN estimates of incidence and mortality worldwide for 36 cancers in 185 countries. CA Cancer J. Clin..

[3] Zhou H., Liu Z., Wang Y. (2022). Colorectal liver metastasis: molecular mechanism and interventional therapy. Signal. Transduct. Target. Ther..

[4] Chen X., Kang R., Kroemer G., Tang D. (2021). Broadening horizons: the role of ferroptosis in cancer. Nat. Rev. Clin. Oncol..

[5] Jiang X., Stockwell B.R., Conrad M. (2021). Ferroptosis: mechanisms, biology and role in disease. Nat. Rev. Mol. Cell Biol..

[6] Cui W., Guo M., Liu D. (2024). Gut microbial metabolite facilitates colorectal cancer development via ferroptosis inhibition. Nat. Cell Biol..

[7] Friedmann Angeli J.P., Schneider M., Proneth B. (2014). Inactivation of the ferroptosis regulator Gpx4 triggers acute renal failure in mice. Nat. Cell Biol..

[8] Doll S., Proneth B., Tyurina Y.Y. (2017). ACSL4 dictates ferroptosis sensitivity by shaping cellular lipid composition. Nat. Chem. Biol..

[9] Smeets E., Huang S., Lee X.Y. (2022). A disease-associated missense mutation in CYP4F3 affects the metabolism of leukotriene B4 via disruption of electron transfer. J. Cachexia Sarcopenia Muscle.

[10] Kikuta Y., Kusunose E., Endo K. (1993). A novel form of cytochrome P-450 family 4 in human polymorphonuclear leukocytes. cDNA cloning and expression of leukotriene B4 omega-hydroxylase. J. Biol. Chem..

[11] (2024,). Stelzer - 2016 - Current Protocols in Bioinformatics.

[12] Wang B., Ge S., Wang Z. (2023). Analysis and experimental validation of fatty acid metabolism-related genes prostacyclin synthase (PTGIS) in endometrial cancer. Aging.

[13] Jia Y., Liu Y., Han Z., Tian R. (2021). Identification of potential gene signatures associated with osteosarcoma by integrated bioinformatics analysis. PeerJ..

[14] Zhao M., Li M., Chen Z. (2020). Identification of immune-related gene signature predicting survival in the tumor microenvironment of lung adenocarcinoma. Immunogenetics.

[15] Ahmed K.M., Veeramachaneni R., Deng D. (2022). Glutathione peroxidase 2 is a metabolic driver of the tumor immune microenvironment and immune checkpoint inhibitor response. J. Immunother. Cancer.

[16] Tang Z., Kang B., Li C., Chen T., Zhang Z. (2019). GEPIA2: an enhanced web server for large-scale expression profiling and interactive analysis. Nucleic Acids Res..

[17] Chandrashekar D.S., Karthikeyan S.K., Korla P.K. (2022). UALCAN: an update to the integrated cancer data analysis platform. Neoplasia.

[18] Tandon N., Thakkar K.N., LaGory E.L., Liu Y., Giaccia A.J. (2018). Generation of stable expression mammalian cell lines using lentivirus. Bio Protoc..

[19] Győrffy B. (2024). Transcriptome-level discovery of survival-associated biomarkers and therapy targets in non-small-cell lung cancer. Br. J. Pharmacol..

[20] Dodson M., Castro-Portuguez R., Zhang D.D. (2019). NRF2 plays a critical role in mitigating lipid peroxidation and ferroptosis. Redox. Biol..

[21] Kensler T.W., Wakabayashi N. (2010). Nrf2: friend or foe for chemoprevention?. Carcinogenesis.

[22] Ma Q., He X. (2012). Molecular basis of electrophilic and oxidative defense: promises and perils of Nrf2. Pharmacol. Rev..

[23] Ma Q. (2013). Role of nrf2 in oxidative stress and toxicity. Annu. Rev. Pharmacol. Toxicol..

[24] O'Malley J., Kumar R., Inigo J., Yadava N., Chandra D. (2020). Mitochondrial stress response and cancer. Trends Cancer.

[25] Rojo de la Vega M., Chapman E., Zhang D.D (2018). NRF2 and the hallmarks of cancer. Cancer Cell.

[26] Bae T., Hallis S.P., Kwak M.K. (March 1, 2024). Hypoxia, oxidative stress, and the interplay of HIFs and NRF2 signaling in cancer. Exp. Mol. Med..

[27] Wu J., Minikes A.M., Gao M. (2019). Intercellular interaction dictates cancer cell ferroptosis via NF2-YAP signalling. Nature.

[28] Stockwell B.R., Jiang X., Gu W. (2020). Emerging Mechanisms and disease relevance of ferroptosis. Trends Cell Biol..

[29] Christmas P., Ursino S.R., Fox J.W., Soberman R.J. (1999). Expression of the CYP4F3 gene. tissue-specific splicing and alternative promoters generate high and low K(m) forms of leukotriene B(4) omega-hydroxylase. J. Biol. Chem..

[30] Christmas P., Jones J.P., Patten C.J. (2001). Alternative splicing determines the function of CYP4F3 by switching substrate specificity. J. Biol. Chem..

[31] McGiff J.C., Carroll M.A. (1991). Cytochrome P450-dependent arachidonate metabolites, renal function and blood pressure regulation. Adv. Prostaglandin Thromboxane Leukot. Res..

[32] Zhang C.Y., Zhong W.J., Liu Y.B. (2023). EETs alleviate alveolar epithelial cell senescence by inhibiting endoplasmic reticulum stress through the Trim25/Keap1/Nrf2 axis. Redox Biol..

[33] Wen J., Aili A., Yan Y.X. (2022). OIT3 serves as a novel biomarker of hepatocellular carcinoma by mediating ferroptosis via regulating the arachidonic acid metabolism. Front. Oncol..

[34] Costa T.J., Ceravolo G.S., Echem C. (2018). Detrimental effects of testosterone addition to estrogen therapy involve cytochrome P-450-induced 20-HETE synthesis in aorta of ovariectomized spontaneously hypertensive Rat (SHR), a model of postmenopausal hypertension. Front. Physiol..

[35] Alexanian A., Rufanova V.A., Miller B., Flasch A., Roman R.J., Sorokin A. (2009). Down-regulation of 20-HETE synthesis and signaling inhibits renal adenocarcinoma cell proliferation and tumor growth. Anticancer Res..

[36] Dhanasekaran A., Bodiga S., Gruenloh S. (2009). 20-HETE increases survival and decreases apoptosis in pulmonary arteries and pulmonary artery endothelial cells. Am. J. Physiol. Heart. Circ. Physiol..

[37] Borin T.F., Angara K., Rashid M.H., Achyut B.R., Arbab A.S. (2017). Arachidonic Acid Metabolite as a Novel Therapeutic Target in Breast Cancer Metastasis. Int. J. Mol. Sci..

[38] Sivinski J., Zhang D.D., Chapman E. (2021). Targeting NRF2 to treat cancer. Semin. Cancer Biol..

[39] Adinolfi S., Patinen T., Jawahar Deen A. (2023). The KEAP1-NRF2 pathway: targets for therapy and role in cancer. Redox Biol..

