# Supplementary material for: Cytochrome P450 F3 promotes colorectal cancer via inhibiting NRF2-mediated ferroptosis
Source: Transl Oncol. 2024 Aug 5;48:102077. doi: 10.1016/j.tranon.2024.102077 (PMC11357859; doi:10.1016/j.tranon.2024.102077)

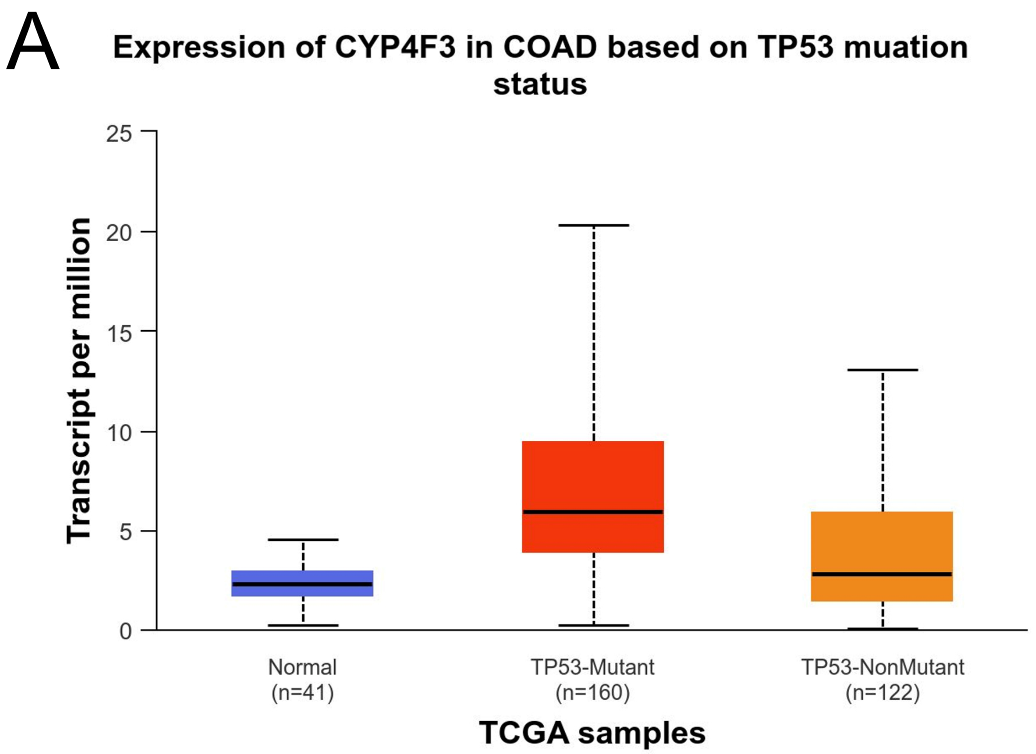

Comparison	P-Value
Normal-vs-TP53-Mutant	**1.25×10^{-6}**
Normal-vs-TP53-NonMutant	9.77×10^{-2}
TP53-Mutant-vs-TP53-NonMutant	**2.98×10^{-8}**

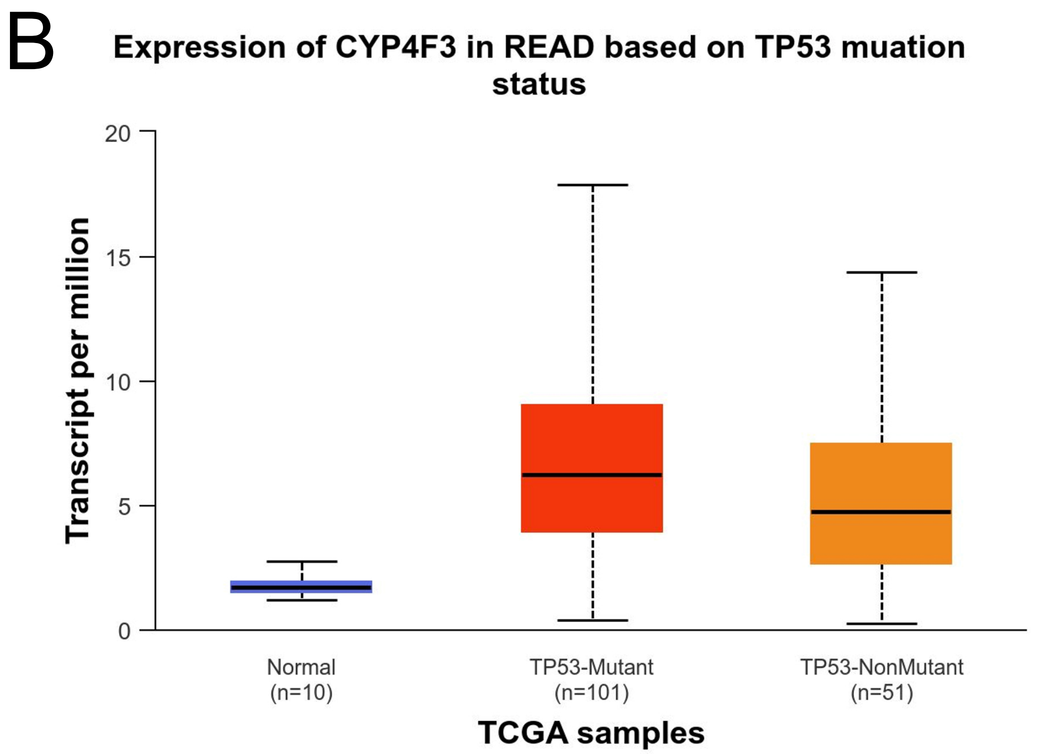

Comparison	P-Value
Normal-vs-TP53-Mutant	**1.62×10^{-12}**
Normal-vs-TP53-NonMutant	**1.77×10^{-8}**
TP53-Mutant-vs-TP53-NonMutant	**3.98×10^{-4}**

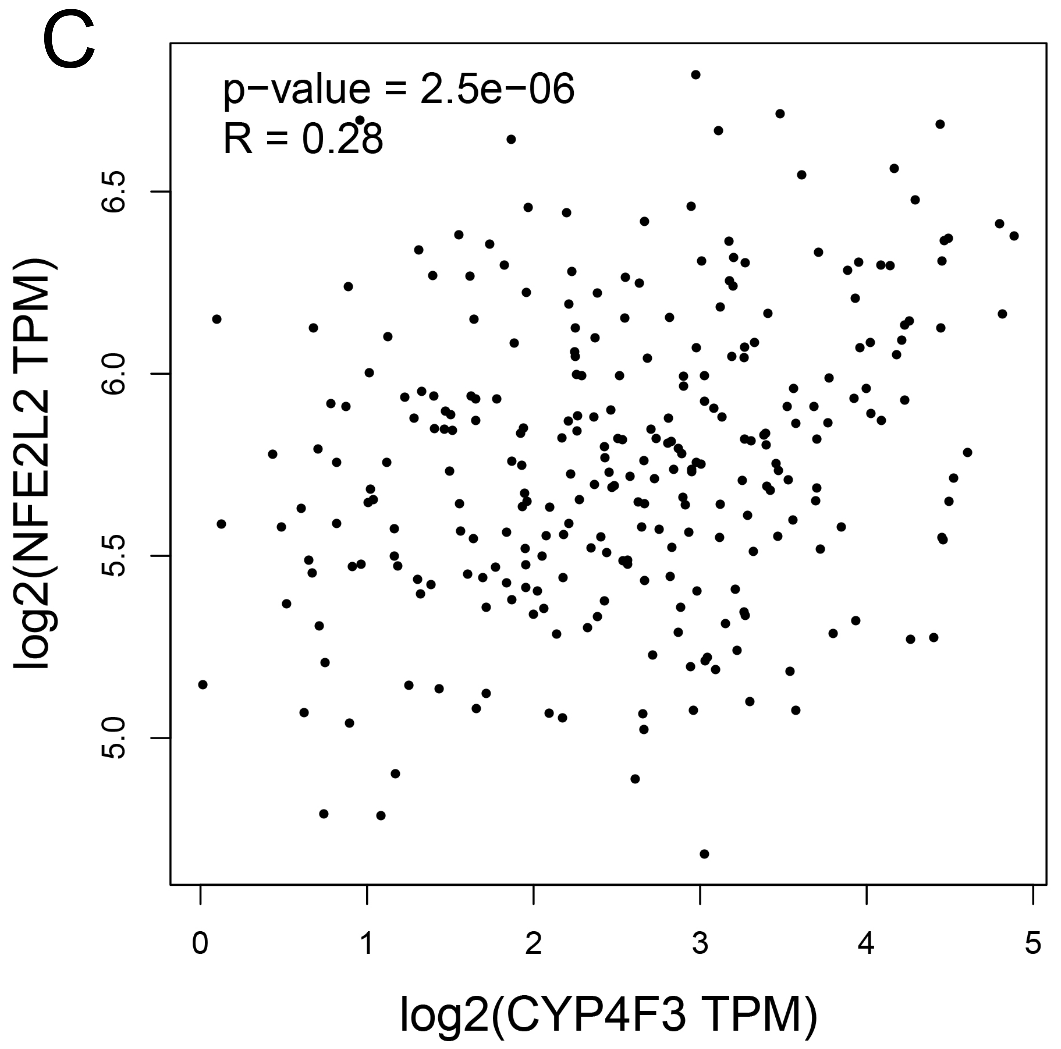

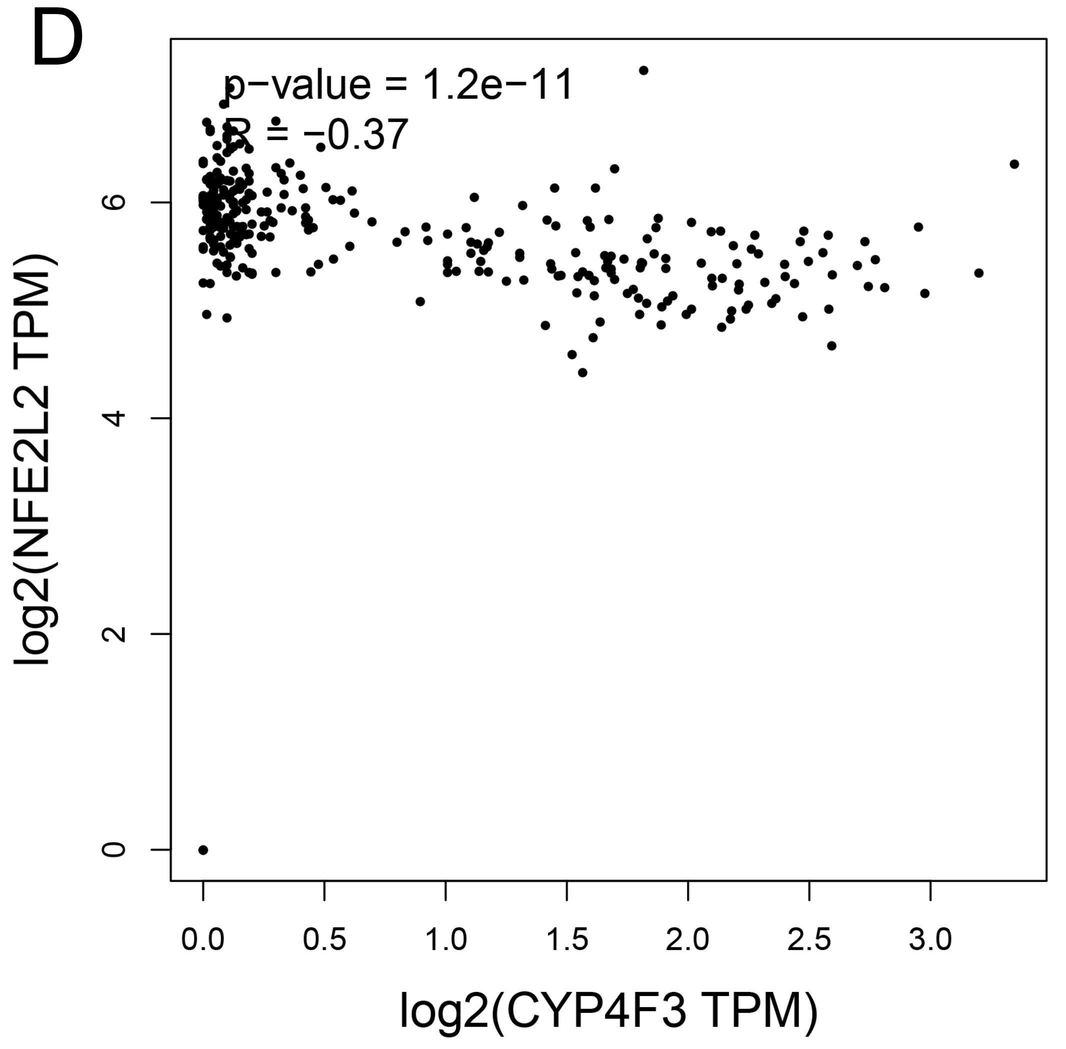

Supplement: Supplementary file 1 [file mmc1.pdf]
