# Supplementary material for: Cytochrome P450 F3 promotes colorectal cancer via inhibiting NRF2-mediated ferroptosis
Source: Transl Oncol. 2024 Aug 5;48:102077. doi: 10.1016/j.tranon.2024.102077 (PMC11357859; doi:10.1016/j.tranon.2024.102077)
Supplement: Supplementary file 2 [file mmc2.docx]

Table 1. Primer sequences of qRT-PCR

| **Gene** | **Forward primer (5’-3’)** | **Reverse primer (5’-3’)** |
| --- | --- | --- |
| **CYP4F3** | ATTGGTTCTTGGGTCACCTG | CCACCAGCAGCACATATCAC |
| **Il-1** | CTCGCAGCAGCACATCAACAAG | CCACGGGAAAGACACAGGTAGC |
| **Tnf-α** | ACGTGGAACTGGCAGAAGAG | CTCCTCCACTTGGTGGTTTG |
| **Nox4** | GAAAACCTTCCTGCTGTACAAC | CTGCTTAAACACAATCCTAGGC |
| **Nrf2** | GTTGCCACCGCCAGGACTAC | GTGCTCAGAAACCTCCTTCCAAAAC |
| **GAPDH** | CGTGCCGCCTGGAGAAAC | AGTGGGAGTTGCTGTTGAAGTC |

**Supplemental figure legends**

Supplemental Figure S1. Relationship of CYP4F3 with TP53 Mutation and NRF2 Expression in CRC. A-B. Association between CYP4F3 expression and TP53 mutation status in CRC; C. Analysis using GEPIA2 reveals a positive correlation between CYP4F3 expression and NRF2 levels in CRC tumor tissues from TCGA dataset; D. Analysis using GEPIA2 demonstrates a significant negative correlation between CYP4F3 expression in normal intestinal tissues from TCGA and GTEx datasets and NRF2 expression.
